# Supplementary material for: Association between oral microbiome and seven types of cancers in East Asian population: a two-sample Mendelian randomization analysis
Source: Front Mol Biosci. 2023 Nov 21;10:1327893. doi: 10.3389/fmolb.2023.1327893 (PMC10702768; doi:10.3389/fmolb.2023.1327893)
Supplement: Supplementary file 1 [file DataSheet1.ZIP › Supplementary Materials/MR plots for tongue/tongue═╝/Lung cancer/pheno.237_to_lung cancer_forest.pdf]

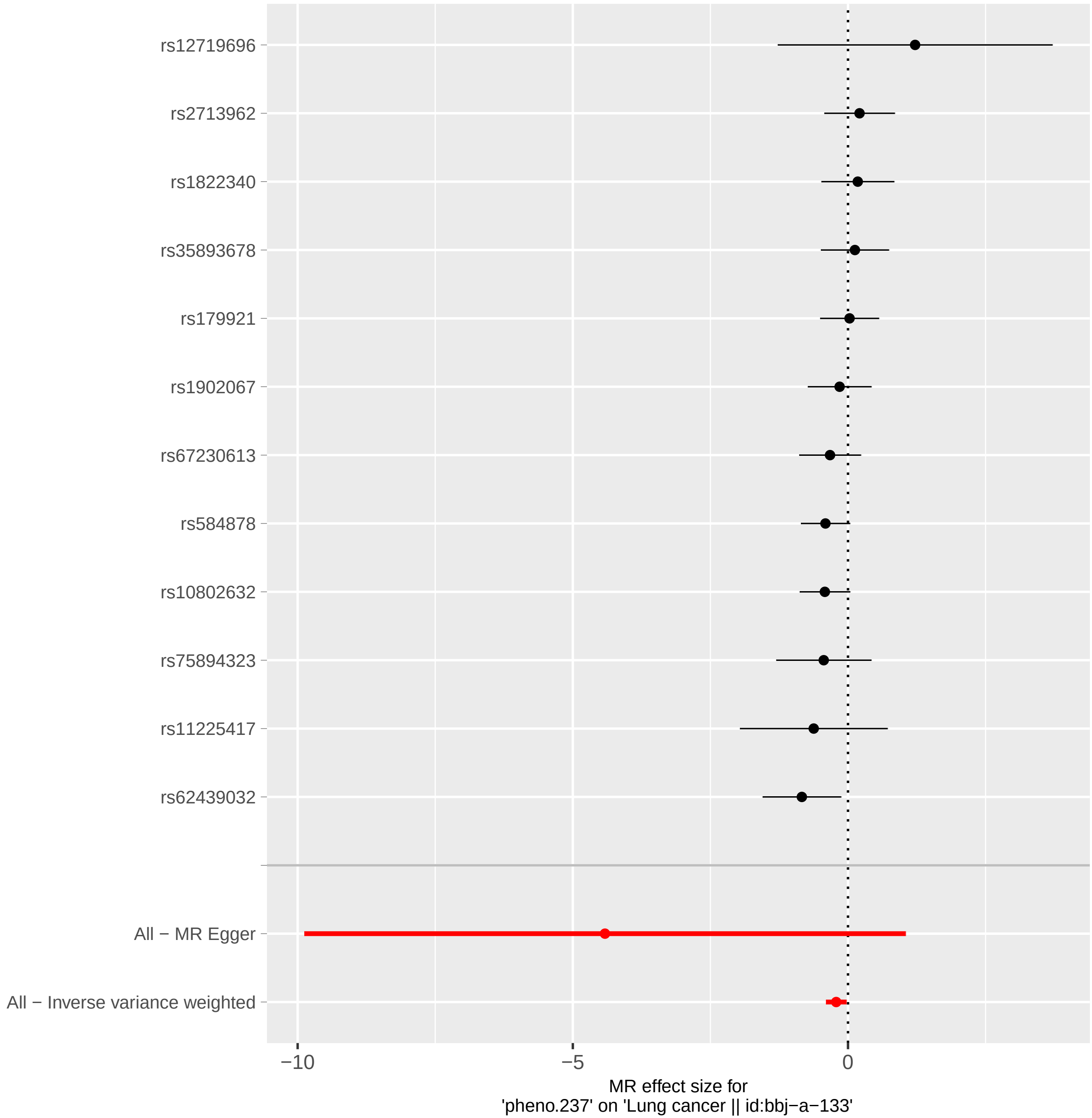

rs12719696
rs2713962
rs1822340
rs35893678
rs179921
rs1902067
rs67230613
rs584878
rs10802632
rs75894323
rs11225417
rs62439032
All − MR Egger
All − Inverse variance weighted
−10
−5
0
MR effect size for
'pheno.237' on 'Lung cancer || id:bbj−a−133'
